# Demand for Long-Term Care Insurance in China

**DOI:** 10.3390/ijerph15010006

**Published:** 2017-12-22

**Authors:** Qun Wang, Yi Zhou, Xinrui Ding, Xiaohua Ying

**Affiliations:** 1 Faculty of Humanities and Social Sciences, Dalian University of Technology, Dalian 116024, China; qunwang@dlut.edu.cn (Q.W.); dxr00329@mail.dlut.edu.cn (X.D.); 2 School of Public Health, Fudan University, Shanghai 200032, China; 15111020036@fudan.edu.cn

**Keywords:** demand, long-term care insurance, China

## Abstract

The aim of this study was to estimate willingness to pay (WTP) for long-term care insurance (LTCI) and to explore the determinants of demand for LTCI in China. We collected data from a household survey conducted in Qinghai and Zhejiang on a sample of 1842 households. We relied on contingent valuation methods to elicit the demand for LTCI and random effects logistic regression to analyze the factors associated with the demand for LTCI. Complementarily, we used document analysis to compare the LTCI designed in this study and the current LTCI policies in the pilot cities. More than 90% of the respondents expressed their willingness to buy LTCI. The median WTP for LTCI was estimated at 370.14 RMB/year, accounting for 2.29% of average annual per capita disposable income. Price, age, education status, and income were significantly associated with demand for LTCI. Most pilot cities were found to mainly rely on Urban Employees Basic Medical Insurance funds as the financing source for LTCI. Considering that financing is one of the greatest challenges in the development of China’s LTCI, we suggest that policy makers consider individual contribution as an important and possible option as a source of financing for LTCI.

## 1. Introduction

The Chinese population is ageing and will continue to age dramatically. The United Nations projects that the percentage of Chinese people aged 60 years or above was 12.4% (168 million people) in 2010 and will increase to 28% (402 million) by 2040 [1]. The pace of population aging in China is much faster than that in other developed and developing countries [2]. Together with this demographic change, China is simultaneously witnessing great socioeconomic transition. Accelerated by the one-child policy, the so-called 4-2-1 family structure (i.e., a family constituted by four grandparents, two parents and one child) has become the main stream family structure in urban China [3]. This socioeconomic transition weakens the traditional familial duties of caring and supporting the elderly [4]. As a result, a huge number of elderly Chinese are now choosing to live alone [5]. Given that long-term care is quite costly in developed countries [6], the above demographic and socioeconomic shift makes the financing of long-term care a significant concern for policymakers in China.

Long-term care insurance (LTCI) is acknowledged as the most desirable policy choice among the existing public financing models of long-term care in China [7], which is also highly recommended for a middle-income country familiar with the public insurance system for the financing of long-term care [8]. In June 2016, the Ministry of Human Resources and Social Security in China issued a document “Guidance on Pilot Cities to Launch Long-Term Care Insurance”, which signified the official initiation of LTCI in China. A total of 15 cities were designated as pilot cities. Based on the experience in the pilot cities, China aims to formally design the policy framework of LTCI by 2020. The policy recommendations made for pilot cities to design LTCI’s target participants, eligible criteria, financing mechanism, and benefit package include the following focal points: (1) The participants of LTCI are in principle those covered by Urban Employees Basic Medical Insurance (UEBMI), a public health insurance covering the employed urban population; (2) It is the severely disabled to whom LTCI mainly provides financial protection; (3) In the pilot stage, it suggested that LTCI raise funds by optimizing the structure of UEBMI funds, transferring UEBMI pooled funds, and adjusting the contribution rate of UEBMI and so on; (4) LTCI is advised to pay 70% of the costs that meet the requirements of reimbursement; (5) Pilot cities are encouraged to gradually expand participation and relax the restrictions on eligibility based on their own circumstances, to explore the multi-channel financing mechanism step by step, and to enhance the benefit package on a gradual basis according to the development of the economy [9].

One of the greatest challenges in the development of China’s LTCI is the mobilization of sufficient funds [10,11]. In the trial run, the financing channel of UEBMI funds, which is heavily emphasized in the guidance, is convenient for China to gain experience in LTCI. However, it is not a sustainable channel [10,11]. Therefore, the information on how much the participants are willing to pay for LTCI is crucial for Chinese policy makers to design a sustainable financing policy for LTCI.

Two methods are usually used to estimate willingness to pay (WTP): a revealed preference approach and a stated preference approach. The former is based on actual choices. The latter is based on hypothetical choices in surveys and is widely used to estimate the monetary value of a non-marketed commodity, such as health care [12]. The contingent evaluation method (CVM) is one of the most frequently used stated preference approaches [13]. With regard to health insurance, revealed preferences data can only be obtained from post-scheme design studies. Thus such studies are rarely used to give policy implications in the design of the schemes [14]. In low- and middle-income countries, a large number of papers have been published relying on CVM to elicit WTP of the proposed health insurance, aiming at informing policy makers about the financing design of the schemes [15,16,17,18,19,20,21,22,23], among which several were based in China [22,23].

However, we could identify only a small number of papers focusing on WTP for LTCI globally. Some researchers relied on CVM to estimate WTP for LTCI in Japan [24] and Spain [25]. Other used a discrete choice experiment, another stated preference approach, to explore WTP for LTCI in Italy [26] and the U.S. [27]. However, until now, no papers have been published on WTP for LTCI in China. Only two papers were identified that were related to LTCI in China. One paper qualitatively evaluated the emerging models to finance long-term care for the aging population [7]. The other was concerned with the factors associated with the preference for public or private LTCI plans and the level of the appropriate premium of each plan [28]. The latter paper did not focus on how much respondents were willing to pay for LTCI, but on the premium that they consider appropriate, which is different from their WTP. In addition, the latter paper did not describe the benefit package of LTCI plans. This limitation affects the results from that paper since respondents were asked to express their views on the appropriate premium of LTCI plans without knowing what these plans offered them.

This study aims at filling this gap by estimating WTP for LTCI in China using CVM and exploring the determinants of demand for LTCI in China.

## 2. Materials and Methods

### 2.1. Data and Data Collection

This LTCI study was a part of a larger project on the public health insurance system in China. We obtained ethical approval from the ethical committee of the School of Public Health, Fudan University, China (Ethical Approval Code: IRB#2010-03-0226). Oral informed consent was received from each participant in the study. In the summer of 2010, we conducted a household survey to collect data in Qinghai Province in western China, and Zhejiang Province in eastern China. We used a two-stage stratified sampling method to obtain our sample. In the first stage, one or two cities were selected in each province. In the second stage, districts were selected in each city. Overall, we investigated 901 and 941 households in Qinghai and Zhejiang, respectively. The heads of the 1842 households were asked about their WTP for LTCI. A total of 1743 heads provided usable answers. In 2010, the mean annual per capita disposable income of urban residents in the respective cities in Qinghai (Xining) and in Zhejiang (Wenzhou and Taizhou) was 14,085 RMB, 27,250 RMB and 27,212 RMB, respectively [29]. The city in Qinghai is the provincial capital with the highest level of economic development in this province. The two cities in Zhejiang both have an above-average level of economic development in this province. Other information collected in the household survey included demographic background, household and family information, health status, and health care utilization.

### 2.2. Household Interview

We developed our WTP questionnaire based on a series of methods, including a thorough document analysis of LTCI policies, especially the public LTCI policies around the world, in-depth interviews with several policy makers in health and social security bureaus, and a pre-testing (i.e., the correction of an ambiguous wording problem). The hypothetical LTCI designed in this study would pay 75% of the total expenses of long-term care, which would be provided at homes or in institutions and in line with a person’s health status for those aged 65 or above and in need of long-term care. In this study, we used the bidding game with follow-up questions to elicit WTP. In the household interview, the designed LTCI was first described to the respondents. Then the WTP questions were asked. For example, ‘Are you willing to buy this LTCI if its annual premium is 350 RMB?’ Eight bids were designed in this study, ranging from 100 RMB to 800 RMB. The starting bid was set at 350 RMB in this study. Follow-up questions varied in order to elicit the highest amount that a household head was willing to pay. If the household head was still willing to buy the insurance at the bid up to 800 RMB or down to 100 RMB, then an open-ended question was asked to avoid truncation.

### 2.3. Regression Analysis

Logistic regression was used to estimate the median WTP and to study the determinants of demand for LTCI [30]. Being multiple bounded bids by nature, we used a person’s responses to all eight bids as separate draws, and thus the sample size was expanded by eight times. This method is strongly recommended for the analysis of WTP data since the sample size is enlarged and thus the efficiency of welfare estimates is increased [31]. In line with our previous study on WTP for private health insurance in China [22] and other studies [31,32], we used the model with random effects to adjust for clustering between the multiple answers provided by the same respondent. In this study, logistic regression with random effects is expressed as follows:

(1) $$\mathrm{Log}\left(\frac{P_{ij}}{1-P_{ij}}\right)=\alpha_{0}+\beta_{1}Price_{ij}+b_{1}X_{1j}\ldots +b_{n}X_{nj}+u_{i}+e_{ij}$$

where *P_ij_* is the probability of the individual *j* buying LTCI at the bid of *i*, α_0_ is the intercept, *Price_ij_* represents the bid of *i* that the individual *j* faces, *X_n_* are the independent variables other than price, *β*_1_ is the coefficient of price, *b_i_* are the coefficients of *X_n_*, *u_i_* is the error term independent across individuals, and *e_ij_* is the error term independent both across individuals and bids and is also independent of *u_i_*. The median WTP = $-\alpha_{0}/\beta_{1}$. The variance of *u_i_* is denoted as $\delta_{u}^{2}$ and the variance of *e_ij_* is denoted as $\delta_{e}^{2}$. The intraclass correlation coefficient (ICC) is calculated as the variance of *u_i_* $\left(\delta_{u}^{2}\right)$ divided by the total variance $\left(\delta_{u}^{2}+\delta_{e}^{2}\right)$. In this study, ICC was used to indicate the dependency between the multiple answers from the same respondent. Rho was utilized as an estimation of ICC.

Cameron proposed a direct way of estimating the median WTP from logit or probit models [33]. The probability *P_ij_* is set at 0.5, yielding the median WTP = $-\alpha_{0}/\beta_{1}$.

We used the Wald test to check the statistical significance of the fixed parameters in the model and the likelihood ratio test to check whether our model with random effects was significantly different from the model without random effects.

Independent variables in this study included price, province, gender, age, marriage status, education, annual per capita income, the type of public health insurance, and whether the respondent was suffering from chronic diseases (Table 1). Two variables, namely the type of public health insurance and annual per capita income, need further explanation. Three public health insurance schemes coexist in China: UEBMI—established in 1998; Urban Residents Basic Medical Insurance (URBMI)—established in 2007 and covering the unemployed urban population; and New Cooperative Medical Scheme (NCMS)—established in 2003 and covering rural population [7]. These three public health insurance schemes are different in terms of their participants, financing mechanisms, and benefit packages (Appendix A, Table A1) [34]. In recent years, URBMI and NCMS have been merged together into Basic Medical Insurance for Urban and Rural Residents (BMIURR) in many places in China [35]. Considering the very small percentage of respondents covered by NCMS in this study and the integration of URBMI and NCMS in recent years, we categorized those with URBMI and with NCMS into one group. We need to point out that at the time of data collection in this study, URBMI and NCMS were still two separate public health insurances in the study settings. In addition, annual per capita income was calculated as the annual household disposable income divided by the number of household members.

### 2.4. Document Analysis

Complementarily, since we conducted our data on WTP for LTCI prior to the official initiation of LTCI in China, we presented an overview on the current LTCI policies in the pilot cities in the hope that the readers can better understand the policy implications of this study. Among the 15 pilot cities listed in the guidance, 14 have already issued LTCI plans (as of mid-October, 2017), and the related documents were the main analyzed materials in this study (Appendix A, Table A2). In the case that anything was unclear or absent from the documents, we reviewed the grey literature (e.g., online/newspaper articles or scientific publications), or consulted the relevant government staff by phone. The information extracted from the documents was participants, eligible criteria, financing mechanism, benefit package, and the time that the current policies came into effect.

## 3. Results

### 3.1. Sample Characteristics

Table 2 shows the characteristics of the sample in this study. Of the 1743 respondents, 52% were from Qinghai, 62% were male, and 74% were married. More than half of the respondents were aged from 35 to 65, while the proportions aged 35 or below and 65 or above were 21% and 23%, respectively. Nearly 50% of the entire respondents had secondary education, much higher than the proportions of those with tertiary education (20%) and those with primary education or below (31%). 29%, 22% and 0.3% of the respondents were covered by UEBMI, URBMI, and NCMS, respectively. 47% of the respondents did not know the type of public health insurance they had. The rest, 1.5%, did not have any public health insurance. Most of the respondents did not suffered from any chronic diseases. Additionally, people in the lowest income quartile had an average annual per capita income of 3473 RMB, while the average annual per capita income in the highest quartile was 36,233 RMB, more than 10 times than that in the lowest quartile.

### 3.2. Mean and Median WTP for LTCI

**Figure 1:**
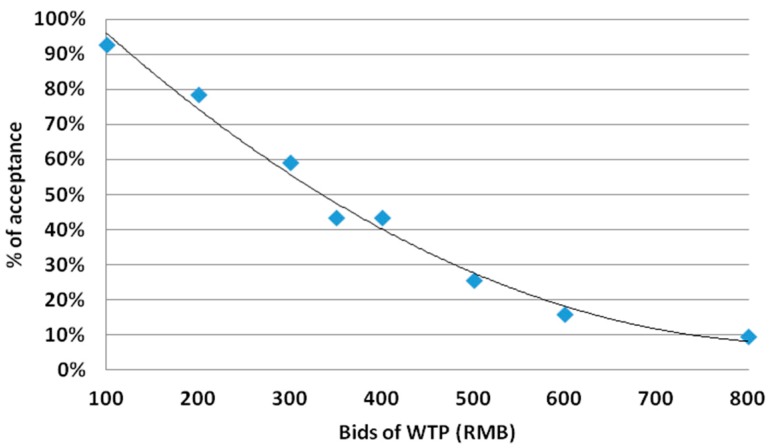
Acceptance rate (%) and bids (RMB).

Mean individual WTP for LTCI was 329.94 RMB/year (Table 2). Among the 1743 respondents, only 6.2% of individuals were not willing to buy LTCI at any price. When individual responses to all eight bids were seen as separate draws, one could clearly see that the likelihood of accepting the bids decreased as bids of WTP increased (Figure 1). Based on the random effects logistic model, the median WTP for LTCI was estimated at 370.14 RMB/year (Table 3), accounting for 2.29% of average annual per capita income among the respondents.

### 3.3. Determinants of Demand for LTCI

Table 3 shows the results of the random effects logistic regression. The coefficients, standard errors, and *p*-values shown in Table 3 were obtained after adjusting for clustering of the outcomes provided by the same individual. The significant factors that were associated with the demand for LTCI were price, age, education status, and income. A higher price was significantly associated with a lower probability of participating in LTCI (*p* < 0.01). Compared to the elderly (65 or above), individuals aged 35 or below were more likely to buy LTCI (*p* < 0.05). Compared to the respondents with tertiary education, those with primary education or below and with secondary education were both less likely to participate in LTCI (both *p* < 0.01). Income significantly increased the demand for LTCI, with individuals both in the higher and highest income quartiles being more likely to buy LTCI than those in the lowest quartile (both *p* < 0.01).

### 3.4. Analysis of the Current LTCI Policies in the Pilot Cities

Among the 14 pilot cities that have issued LTCI plans, three cities have already started LTCIs officially before the central government’s guidance was issued, while the remaining 11 set up LTCIs afterwards. All the 14 cities included UEBMI participants in the LTCIs. Seven cities included URBMI or BMIURR participants as well. Ten cities restricted the LTCI eligibilities to those rated as severely disabled. Only in four cities were the partially disabled also eligible to enjoy the benefits of LTCI. The vast majority of pilot cities mainly relied on the transfer of UEBMI funds as the financing channel. In just four pilot cities, participants paid an individual contribution, ranging from 10 RMB/person/year to 30 RMB/person/year. The related participants of LTCI in each city paid the same amount of individual contribution, which was set by the pilot city according to its own circumstances and was not dependent on a person’s income or the risk of being disabled. In addition, seven cities provided government subsidies to all or some of the participants of LTCI. Concerning the benefit packages, the schemes in 13 pilot cities either paid 50% to 90% of the costs with a relatively low payment ceiling, or paid a limited fixed amount. The payment ceilings usually ranged from 20 RMB/person/day to 70 RMB/person/day and the fixed amount were usually from 10 RMB/person/day to 40 RMB/person/day. The only LTCI pilot scheme in Zhejiang and Qinghai (the scheme in Ningbo) showed similar characteristics as described above. The details of the financing and benefit packages of the current LTCI policies are listed in Appendix A, Table A3 and Table A4.

## 4. Discussion

This study makes an important contribution to the available literature as it is one of the very few studies exploring WTP for LTCI in China. The main results in this paper were that more than 90% of the respondents expressed their willingness to buy LTCI and the median WTP accounted for 2.29% of average annual per capita disposable income. Our finding is best to be interpreted with the current individual contribution rates for LTCI in other developed countries. For example, in Germany the contribution rate for LTCI (split by employers and employees) is 2.55% for parents with children and 2.8% for childless citizens— if older than 23 years—of gross income [8]; the individual contribution rate is 0.9% of gross income for Japanese adults aged 40–64 [36]. We also found that the vast majority of pilot cities mainly rely on UEBMI funds as the financing source for LTCI. China’s LTCI started with a limited benefit package and strict eligibility rules, which is the recommended way for middle-income countries to establish LTCI [8]. As the guidance from the central government foresees, China will gradually expand LTCI [9]. Our findings are very enlightening, especially since China’s LTCI is in the initial period of development and is facing the challenge of financing sustainability [10,11]. Based on the WTP estimated in this study, we suggest that it is acceptable, from the participants’ perspective, that individual contribution is considered an important source of mobilizing funds for LTCI in China. We also demonstrated that among different age groups, the older population (65 or above) revealed less demand for LTCI than the younger population (35 or below), but no significant difference from the population in the middle age group (35 to 64). These results are similar to one systematic review on the demand for health insurance indicating that age was negatively correlated with WTP for health insurance [37] and similar to the previous study showing that older individuals in the U.S were no more likely to reveal a higher possibility of buying LTCI [27]. However, these results are contradictive to the previous study based in Spain revealing that age was positively associated with the demand for LTCI [26]. Generally, the older an individual is, the higher the likelihood that one could benefit from LTCI at average premiums. However, since the younger population was born when the one-child policy was strictly implemented in China, they may already realize the importance of LTCI for older and disabled people and thus display a high demand for LTCI. At the time being, there is a heated debated concerning the starting age of collecting individual contributions for LTCI in China [10,11]. Considering that the demand for LTCI is relatively high among the younger population, we suggest that it is feasible and reasonable to collect the individual contribution for LTCI at a young age.

In line with previous studies on demand for LTCI [24,26,27] and on health insurance [15,17,18,20,21,22,23], we found that the demand for LTCI increased as income, a proxy of ability to pay, increased. The findings that demand for LTCI among the lowest income quartile was quite low were worrisome since this population segment is already the most vulnerable group. Additionally, deprivation of the social protection gained from LTCI will worsen their social-economic status and may let them fall into deeper poverty. These findings suggest that the Chinese government needs to provide proper subsidies to the low-income population who cannot afford the insurance premium. In addition, the positive correlation detected between education and the demand for LTCI aligns with the previous evidence on the demand for LTCI [26] and on health insurance [15,18,20,21,22]. The findings could be attributed to the fact that individuals with higher education usually have a stronger awareness of the actual risk of the costs induced by future long-term care.

Another important finding in this study was that the demand for LTCI in Qinghai (located in western China) showed no significant difference from that in Zhejiang (located in eastern China). China has started to establish LTCI even though China as a whole lacks long-term care providers [7]. LTCI can serve as a stimulus for the development of long-term care delivery systems in middle-income countries [8]. However, a long-term care delivery system will be more difficult to develop in western provinces than in eastern provinces in China, since the western provinces are less developed. To meet the high demand for LTCI in Qinghai, the government has already made and will in the future make more efforts to strengthen affordable high quality long-term care in such provinces [38]. In contrast with prior studies on the demand for health insurance [18,22], suffering from chronic conditions, a representative of poor health status, was not a significant factor associated with the demand for LTCI. These results indicate that when controlling other variables, such as age, income, education, price and so on, health status is not significantly associated with the demand for LTCI in the settings of this study. However, further studies are needed in order to more deeply explore the relationship between health status and the demand for LTCI in other settings in China.

A few limitations of this study need to be acknowledged. First, the starting bid bias, i.e., the fact that the first bid biases respondents’ answers to the subsequent bids, may incur when using the bidding game method. Considering that the median WTP for LTCI estimated in this study was above the starting bid, respondents’ WTP is possibly biased downwards [22]. The starting bid in this study was not varied due to practical constrains. Further study is needed to account for the effect of the starting bid in the estimation of WTP for LTCI. Second, though this paper was a part of the study on the public health insurance system, the LTCI designed in this study did not clearly point out whether it was public or private. The public LTCI plan was found to be more popular than the private in terms of participation and contribution in China [28]. Thus, it can be inferred that if we had clearly pointed out the public nature of LTCI, the actual WTP would have been even higher than the estimates we obtained from this study. Third, the coefficients from our model can be used to estimate the potential enrollment size of LTCI under different prices for sub-groups defined by the independent variables (e.g., age, gender, income). However, the gap always exists between respondents’ behavior in a hypothetical market and in real life. Thus, more research is needed in order to set an appropriate premium for LTCI in China. Fourth, respondents may underreport the annual household income due to recall bias. So the ratio of an individual’s WTP for LTCI to the annual per capita income may be biased upwards. Fifth, we used literature review and interviews with key policy makers to develop our WTP questionnaire. Thus, the hypothetical LTCI designed in this study did not reflect the expectations of the potential participants of LTCI. Further study is needed in order to incorporate community preferences for LTCI into related studies. Meanwhile, the design of the hypothetical LTCI in this study did not account for the government subsidy. Currently, half of LTCI schemes do not include government subsidy as the financing source of LTCI. However, further study is still needed to understand how respondents’ attitude towards government subsidies in LTCI. In addition, comparing the LTCI designed in this study with the current LTCI schemes in pilot cities, our LTCI had a larger benefit package and broader eligibility since it featured similar copayment as those in the current schemes, but without a payment ceiling and without eligibility in terms of the severity of disability. Though our data was collected in 2010, we still believe that our findings have great policy implications for the development of LTCI in China; China’s LTCI will gradually expand its benefit package and relax its restrictions on eligibility, but the fact remains that it is experimenting with a suitable sustainable financing mechanism [10,11]. However, one needs to be cautious in applying the median WTP for LTCI estimated in this study into reality, since the average per capita income, consumer price index and so on are rising year by year. Last, this study addressed the question of whether respondents were willing to buy a designed LTCI under different bids. However, in reality the decision on whether to invest in LTCI is related to how households set priorities on household consumption. More study is needed to explore the trade-off between the investment in LTCI and other aspects of household consumption, such as childcare, purchasing property, etc.

## 5. Conclusions

Our study showed that the demand for LTCI in China was high and individuals were willing to pay a considerable amount of individual contribution for LTCI. We also found that the vast majority of the current LTCI schemes mainly rely on the transfer of UEBMI funds as the financing source. Considering that financing is one of the greatest challenges in the development of China’s LTCI, we suggest that policy makers consider individual contribution as an important and possible option as a source of financing for LTCI. In addition, the estimated WTP value and determinants of demand for LTCI identified in our study can be used to set a proper premium for LTCI in China. However, further studies are still needed with respect to pricing LTCI in China.

## Figures and Tables

**Table 1:** Definitions of variables.

Variable	Measurement
Price of LTCI (RMB)	Ordinal variable
Province	0 = Zhejiang, 1 = Qinghai
Sex	0 = female, 1 = male
Age	
≤35	0 = No, 1 = Yes
35–65	0 = No, 1 = Yes
≥65	65 or above was the default variable
Marital status	0 = Single, divorced, or widowed
	1 = Married
Education status	
Primary education or below	0 = No, 1 = Yes
Secondary education	0 = No, 1 = Yes
Tertiary education	Tertiary education was the default variable
Average annual per capita income (RMB)	
Lowest 25%	Lowest 25% average annual per capita income was the default variable
Middle 25%	0 = No, 1 = Yes
Higher 25%	0 = No, 1 = Yes
Highest 25%	0 = No, 1 = Yes
The type of public health insurance	0 = Not covered or did not know the type of public health insurance that the respondent had
UEBMI	UEBMI was the default variable
URBMI/NCMS	0 = No, 1 = Yes
Not covered or did not know the type	0 = No, 1 = Yes
Having chronic conditions	0 = No, 1 = Yes

**Table 2:** Descriptive Statistics for the variables (*N* = 1743).

Variable	Mean	SD
WTP (The highest bid which a person was willing to pay) (RMB/year)	329.94	219.34
Annual per capita income (RMB)		
Lowest 25%	3472.62	1519.34
Middle 25%	7347.68	1009.81
Higher 25%	12,874.09	2614.97
Highest 25%	36,232.52	21,732.23
Variable	N	%
Province		
Qinghai	901	51.69
Zhejiang	842	48.31
Sex		
Male	1075	61.68
Female	668	38.32
Age (year)		
≤35	370	21.23
35–65	972	55.77
≥65	401	23.01
Marital status		
Married	1292	74.13
Single, divorced, or widowed	451	25.87
Education status		
Primary education or below	538	30.87
Secondary education	848	48.65
Tertiary education	357	20.48
The type of public health insurance		
UEBMI	508	29.15
URBMI/NCMS	387	22.20
Not covered or did not know the type	848	48.65
Having chronic conditions		
Yes	621	35.63
No	1122	64.37

**Table 3:** Coefficients of random effects logistic regression (*N* = 13,944).

Variable	Coef	S.E.	*p*-Value
Intercept	24.786 ***	4.143	<0.001
Price of LTC (RMB)	−0.067 ***	0.003	<0.001
Qinghai province	−0.528	2.660	0.843
Male	−0.059	0.835	0.944
Age of HH			
≤35	3.519 **	1.680	0.036
35–65	−0.268	1.240	0.829
Married	0.612	1.040	0.557
Education status of HH			
Primary education or below	−6.122 ***	1.364	<0.001
Secondary education	−4.044 ***	1.239	<0.001
Annual per capita income (RMB)			
Middle 25%	0.036	1.146	0.975
Higher 25%	4.448 ***	1.228	<0.001
Highest 25%	9.988 ***	1.228	<0.001
The type of public health insurance			
URBMI/NCMS	−1.907	1.183	0.107
Not covered or did not know the type	−1.139	2.651	0.667
Having chronic conditions	−0.829	0.836	0.322
Median WTP (RMB)	370.14		
Random effects			
rho coefficient = 0.98; rho S.E. = 0.0012			
Wald χ^2^(14); *P* > χ^2^	779.01		*p* < 0.001
Likelihood ratio test of rho; *P* > χ^2^	4903.94		*p* < 0.001

Significant at *** 1%, ** 5% and * 10%.

## Appendix A

**Table A1:** Overview of the three public health insurance schemes in China.

	UEBMI	URBMI	NCMS
Participants	employed urban population	unemployed urban population	rural population
Financing mechanism	6% of payroll tax on employers +2% employee contribution	government subsidy + individual contribution (paid in a periodic lump sum)	government subsidy + individual contribution (paid in a periodic lump sum)
Benefit package	UEBMI > URBMI > NCMS		

This table was adapted from [34].

**Table A2:** List of the main analyzed documents in this study.

Cities	The Title of Document
Chengde	Opinions on Implementing Long-term Care Insurance for UEBMI Enrollees in Chengde
Changchun	Opinions on Establishing Long-term Care Insurance for the Disabled in Changchun
	Implementation Measures of Long-term Care Insurance for the Disabled in Changchun (on trial)
Qiqihaer	Implementation Scheme of Long-term Care Insurance in Qiqihaer (on trial)
Shanghai	Pilot Measures of Implementing Long-term Care Insurance in Shanghai
Nantong	Opinions on Establishing Long-term Care Insurance in Nantong (on trial)
	The Rules for Implementing Long-term Care Insurance in Nantong
Suzhou	Opinions on Implementing Long-term Care Insurance in Suzhou
Ningbo	Notice on Issuing Pilot Scheme of Implementing Long-term Care Insurance in Ningbo
Anqing	Opinions on Implementing Long-term Care Insurance for UEBMI Enrollees in Anqing
	The Rules for Implementing Long-term Care Insurance for UEBMI Enrollees in Anqing
Shangrao	Implementation Scheme of Long-term Care Insurance in Shangrao
	Procedures of Administrating Long-term Care Insurance in Shangrao
Qingdao	Administrative Measures of Long-term Care Insurance in Qingdao
Jingmen	Measures of Implementing Long-term Care Insurance in Jingmen (on trial)
Guangzhou	Pilot Measures of Implementing Long-term Care Insurance in Guangzhou
Chengdu	Pilot Scheme of Implementing Long-term Care Insurance in Chengdu
	The Rules for Implementing Long-term Care Insurance in Chengdu (on trial)
Shihezi	Opinions on Establishing Long-term Care Insurance in Shihezi (on trial)
	The Rules for Implementing Long-term Care Insurance in Shihezi (on trial)

**Table A3:** Current LTCI policies in the pilot cities in China I.

Cities	Province	Participants	Financing Mechanism
Chengde	Hebei	UEBMI enrollees	Pilot stage: individual contribution(transfer from MSA of UEBMI ^1^, 0.15% of UEBMI premium base ^2^) + UEBMI pooled funds (0.2% of UEBMI premium base) + government subsidy (0.05% of UEBMI premium base)
Changchun	Jilin	UEBMI and URBMI enrollees	UEBMI enrollees: MSA of UEBMI (0.2% of UEBMI premium base) + UEBMI pooled funds (0.3% of UEBMI premium base) + government subsidy (if UEBMI pooled funds are in deficit) URBMI enrollees: URBMI pooled funds (30 RMB/person/year) + government subsidy (if URBMI funds are in deficit)
Qiqihaer	Heilongjiang	UEBMI enrollees	Pilot stage: individual contribution (transfer from MSA of UEBMI, 30 RMB/person/year) + UEBMI pooled funds (30 RMB/person/year)
Shanghai	Shanghai	UEBMI enrollees and BMIURR enrollees aged 60 or above	UEBMI enrollees: Pilot stage: UEBMI pooled funds. After the pilot: individual contribution (0.1% of UEBMI premium base) + employer contribution (1% of UEBMI premium base) BMIURR enrollees: Pilot stage: BMIURR pooled funds. After the pilot: individual contribution (15% of LTCI fundraising totals) + government contribution
Nantong	Jiangsu	UEBMI and BMIURR enrollees	UEBMI enrollees: individual contribution(transfer from MSA of UEBMI, 30 RMB/person/year) + UEBMI pooled funds (30 RMB/person/year) + government subsidy (40 RMB/person/year) BMIURR enrollees: individual contribution (30 RMB/person/year) + BMIURR pooled funds (30 RMB/person/year) + government subsidy (40 RMB/person/year)
Suzhou	Jiangsu	UEBMI and BMIURR enrollees	Pilot stage: UEBMI enrollees: UEBMI pooled funds (70 RMB/person/year) + government subsidy (50 RMB/person/year) BMIURR enrollees: BMIURR pooled funds (35 RMB/person/year) + government subsidy (50RMB/person/year)
Ningbo	Zhejiang	UEBMI enrollees	UEBMI pooled funds
Anqing	Anhui	UEBMI enrollees	Pilot stage: individual contribution (10 RMB/person/year) + UEBMI pooled funds (20 RMB/person/year)
Shangrao	Jiangxi	UEBMI enrollees	Pilot stage: individual contribution (transfer from MSA of UEBMI, 40 RMB/person/year) + employer contribution (30 RMB/person/year) + UEBMI pooled funds (30 RMB/person/year) + government subsidy (if individuals work in public institutions or the companies in financial difficulties)
Qingdao	Shandong	UEBMI and BMIURR enrollees	UEBMI enrollees: UEBMI pooled funds + MSA of UEBMI (0.5% of UEBMI premium base) BMIURR enrollees: BMIURR pooled funds
Jingmen	Hubei	UEBMI and BMIURR enrollees	The LTCI fundraising totals equaled to 0.4% of the annual per capita disposable income in 2015 in Jingmen (about 82 RMB/person/year). Individual contribution (37.5% of LTCI fundraising totals, about 30 RMB/person/year) + UEBMI or BMIURR pooled funds (25% of LTCI fundraising totals) + government subsidy (government provides full subsidy for vulnerable population to pay their individual contribution, accounting for 37.5% of LTCI fundraising totals)
Guangzhou	Guangdong	UEBMI Enrollees	Pilot stage: UEBMI pooled funds (130 RMB/person/year)
Chengdu	Sichuang	UEBMI enrollees	Pilot stage: individual contribution (transfer from MSA of UEBMI, 0.1%, 0.2%, and 0.3% of UEBMI premium base for those aged 40 years and below, those aged between 40 years and the retirement age, and the retired, respectively) + UEBMI pooled funds (0.2% of UEBMI premium base) + government subsidy(only to retired population)
Shihezi	Xinjiang	UEBMI and URBMI enrollees	UEBMI enrollees: UEBMI pooled funds (180 RMB/person/year) + government subsidy (40 RMB/person/year, to those who are aged 60 plus and severely disabled) URBMI enrollees: individual contribution (24 RMB/person/year)+URBMI pooled funds + government subsidy (40 RMB/person/year, to those who are aged 60 plus and severely disabled)

^1^ UEBMI funds are composed of two parts, medical savings account (MSA) and pooled funds. Currently, Chengde, Qiqihaer, Nantong, Shangrao, and Chengdu transferred a part of MSA of UEBMI to pay the individual contribution; ^2^ UEBMI premium base refers to a person’s average monthly salary in the last year. The lower and upper limit of UEBMI premium base equals 60% and 300%, respectively, of the average monthly salary of all employees in the city in the last year.

**Table A4:** Current LTCI policies in the pilot cities in China II.

Cities	Eligibility	Benefit Package	Effective from
Chengde	the severely disabled	Medical facilities: LTCI pays 70% of the costs, with a payment ceiling of 60 RMB/person/day. Care facilities or nursing homes: LTCI pays 70% of the costs, with a payment ceiling of 50 RMB/person/day.	June 2017
Changchun	the severely disabled	Care facilities or nursing homes: LTCI pays 90% of the costs, with a payment ceiling for UEBMI enrollees, and pays 80% with a payment ceiling for URBMI enrollees. Medical facilities: the payment depends on the level of facilities and the type of health insurance	May 2015
Qiqihaer	the severely disabled	Care facilities: LTCI pays 60% of the costs, with a payment ceiling of 30 RMB/person/day. Nursing homes: LTCI pays 55% of the costs, with a payment ceiling of 25 RMB/person/day. Home care: LTCI pays 50% of the costs, with a payment ceiling of 20 RMB/person/day.	Oct 2017
Shanghai	those rated from secondary to sixth level in disability assessment	Home care: LTCI pays 90% of the costs. The times of home care provided each week depends on the person’s rated level in disability assessment. Nursing homes: LTCI pays 85% of the costs. Medical facilities: according to the requirement of UEBMI and BMIURR.	Jan 2017
Nantong	the severely and partially disabled	Medical facilities: LTCI pays 60% of the costs, with a payment ceiling of 50 RMB/person/day for the severely disabled and 10 RMB/person/day for the partially disabled. Nursing homes: LTCI pays 50% of the costs, with a payment ceiling of 40 RMB/person/day for the severely disabled and 10 RMB/person/day for the partially disabled. Home care: LTCI pays 15 RMB/person/day for the severely disabled and 8 RMB/person/day for the partially disabled.	Jan 2016
Suzhou	the severely and partially disabled	Care facilities or nursing homes: LTCI pays 26 RMB/person/day for the severely disabled, and 20 RMB/person/day for the partially disabled. Home care: LTCI pays 30 RMB/person/day for the severely disabled and 25 RMB/person/day for the partially disabled. Medical facilities: according to the requirement of UEBMI and BMIURR.	Oct 2017
Ningbo	the severely disabled	LTCI pays 40 RMB/person/day for the care provided by nursing homes and specialized care facilities.	Dec 2017
Anqing	the severely disabled	Medical facilities: LTCI pays 60% of the costs, with a payment ceiling of 50 RMB/person/day. Nursing homes: LTCI pays 50% of the costs, with a payment ceiling of 40 RMB/person/day. Home care provided by designated care facilities: The payment ceiling is 750 RMB/person/month. Home care provided by non-designated care facilities: LTCI pays 15 RMB/person/day.	Jan 2017
Shangrao	the severely disabled	Home care provided by relatives or a designated person: LTCI provides a small subsidy for the caregiver. Home care provided by care facilities: LTCI pays by service and by per diem. Institutional care: LTCI pays per diem.	Nov 2016
Qingdao	the severely and partially disabled	LTCI pays 90% of the care costs for UEBMI enrollees, 80% for BMIURR enrollees with the first-type individual contribution, and 40% for BMIURR enrollees with the second-type individual contribution. The payment ceiling is 170 RMB/person/day for specialized institutions, 65 RMB/person/day for nursing homes, 50 RMB/person/day for home care. For care facilities in the community, the payment ceiling is 1600 RMB/person/year for UEBMI enrollees and BMIURR enrollees with the first-type individual contribution and 800 RMB/person/year for BMIURR enrollees with the first-type individual contribution.	Jan 2015
Jingmen	the severely disabled	Fulltime home care: LTCI pays 80% of the costs, with a payment ceiling of 100 RMB/person/day. Part-time home care: LTCI pays 40 RMB/person/day. Nursing homes: LTCI pays 75% of the costs, with a payment ceiling of 100 RMB/person/day. Medical facilities: LTCI pays 70% of the costs, with a payment ceiling of 150 RMB/person/day.	Jan 2017
Guangzhou	the severely disabled	Basic daily care: LTCI pays 75% of the costs, with a payment ceiling of 120 RMB/person/day in nursing homes or care facilities, and 90% of the costs, with a payment ceiling of 115 RMB/person/day for home care. Medical care: LTCI pays by service, with copayment, and with a payment ceiling of 1000 RMB/person/month.	Aug 2017
Chengdu	the severely disabled	Institutional care: LTCI pays 70% of the costs, with a payment ceiling ranging from 1005 RMB/person/month to 1676 RMB/person/month. Home care: LTCI pays 70% of the costs, with a payment ceiling ranging from 1077 RMB/person/month to 1796 RMB/person/month.	July 2017
Shihezi	the severely disabled	Designated institutional care: LTCI pays 70% of the costs, with a payment ceiling of 750 RMB/person/month. Non-designated institutional care and home care: LTCI pays 25 RMB/person/day.	Jan 2017
